# Health Care Access Among U.S. Adults Who Drink Alcohol Excessively: Missed Opportunities for Prevention

**Published:** 2006-03-15

**Authors:** Timothy S Naimi, Machell Town, Ali H Mokdad, Robert D Brewer

**Affiliations:** Emerging Investigations and Analytic Methods Branch, Division of Adult and Community Health, National Center for Chronic Disease Prevention and Health Promotion, Centers for Disease Control and Prevention, Zuni PHS Hospital; Behavioral Surveillance Branch, Division of Adult and Community Health, National Center for Chronic Disease Prevention and Health Promotion, Centers for Disease Control and Prevention, Atlanta, Ga; Behavioral Surveillance Branch, Division of Adult and Community Health, National Center for Chronic Disease Prevention and Health Promotion, Centers for Disease Control and Prevention, Atlanta, Ga; Emerging Investigations and Analytic Methods Branch, Division of Adult and Community Health, National Center for Chronic Disease Prevention and Health Promotion, Centers for Disease Control and Prevention, Atlanta, Ga

## Abstract

**Introduction:**

Excessive alcohol consumption kills approximately 75,000 people annually in the United States. Although alcohol screening among primary care patients is recommended by the U.S. Preventive Services Task Force, it is rarely performed. It is unclear whether low screening rates are due to limited access to health care, missed screening opportunities during patient visits, or both.

**Methods:**

Data came from the 2002 Behavioral Risk Factor Surveillance System, a population-based telephone survey of noninstitutionalized U.S. adults. Current health insurance status and a history of a recent medical checkup (within 2 years) were assessed in relation to alcohol consumption status. Excessive drinkers included those who reported binge drinking (consuming five or more drinks on one or more occasions in the past month), heavy drinking (consuming more than 60 drinks in the past month for men or more than 30 for women), or both.

**Results:**

The prevalence of excessive drinking among the general population (17%) was only slightly higher than the prevalence among those with current health insurance (15%) or a recent checkup (14%). Among excessive drinkers, 79% had current health insurance and 78% had a recent checkup. Although excessive drinkers were somewhat less likely to have health insurance or a recent checkup compared with nonexcessive drinkers and nondrinkers, these differences were less pronounced after stratifying by age. Excessive drinkers with the lowest rates of health insurance were young, Hispanic, less educated, and unemployed. However, most excessive drinkers who lacked insurance or a checkup were employed.

**Conclusion:**

Most excessive drinkers were insured and had a recent medical checkup, suggesting that low screening rates among excessive drinkers are mostly due to missed screening opportunities rather than a lack of screening opportunities. Systems approaches to address these missed opportunities should be aggressively implemented.

## Introduction

Excessive alcohol consumption is the third leading "actual" cause of death in the United States (1) and was responsible for 75,000 deaths and 2.3 million years of potential life lost (about 30 years of life lost per death) in 2001 (2). Approximately 30% of current drinkers in the United States drink excessively (3). Excessive drinking refers to per-occasion consumption or average consumption of alcohol that puts individuals at increased risk for alcohol-related health and social problems (4,5). Excessive drinking is associated with a wide range of serious problems such as liver cirrhosis, gastrointestinal cancers, hemorrhagic stroke, heart failure, motor vehicle crashes, interpersonal violence, sexually transmitted diseases, unintended pregnancy, and a variety of neurological problems including fetal alcohol syndrome. Approximately 10% to 15% of excessive drinkers are alcohol-dependent (6).

The U.S. Preventive Services Task Force recommends routine screening of adults for alcohol *misuse* (i.e., excessive drinking) in primary care settings (7). The U.S. Preventive Services Task Force also recommends brief counseling interventions for individuals with nondependent alcohol misuse; brief counseling has been shown to reduce alcohol consumption and alcohol-related harms (7,8). Despite this, few adult primary care patients are screened for alcohol consumption, and alcohol-related screening is one of the least commonly performed of the recommended clinical preventive services (9,10).

One explanation for low rates of screening may be that excessive drinkers have limited access to care, either because of lower rates of health insurance, inability to obtain routine medical checkups, or both. This could, in turn, influence clinicians' beliefs about the usefulness of screening for excessive drinking during routine medical visits. On the other hand, the low rates of screening and intervention for alcohol problems could also reflect a failure of the health care system to appropriately integrate alcohol screening into routine medical care. We used data from the 2002 Behavioral Risk Factor Surveillance System (BRFSS) to assess health care access and the use of clinical preventive services among U.S. adults by alcohol consumption status, including among those who drink excessively.

## Methods

The BRFSS is a nationally representative, population-based series of telephone surveys conducted by state health departments and coordinated by the Centers for Disease Control and Prevention (CDC). The BRFSS uses a random-digit–dialing algorithm to select a representative sample of the noninstitutionalized adult population in the 50 states, Washington, DC, and the territories of Guam, Puerto Rico, and the U.S. Virgin Islands. BRFSS state and territorial data are combined to produce national estimates. There were 246,964 respondents to the BRFSS in 2002, the most recent year for which data on medical checkups were available. (More information about BRFSS methodology is available from www.cdc.gov/brfss/pubs/index.htm#methodologies.)

We defined excessive drinkers as respondents who met BRFSS criteria for binge drinking, heavy drinking, or both. For the 2002 BRFSS, binge drinking was defined as consuming five or more alcoholic beverages on one or more occasions during the past 30 days. Heavy drinking was defined as consuming more than 60 drinks for men or more than 30 drinks for women during the past 30 days. Nonexcessive drinkers were defined as individuals who reported some alcohol consumption during the past 30 days but who did not meet criteria for excessive drinking. Nondrinkers were defined as individuals who reported no alcohol consumption during the past 30 days. After excluding 404 (0.16%) respondents with missing or incomplete alcohol information, 35,176 excessive drinkers, 93,695 nonexcessive drinkers, and 117,689 nondrinkers were available for analysis in this study. BRFSS data were subsequently weighted for age, sex, and race and ethnicity to be representative of the U.S. population. 

Current health insurance status served as a proxy for health care access, and health checkups within 24 months were used to assess the use of clinical preventive services. Health insurance status was assessed by asking, "Do you have any kind of health care coverage, including health insurance, prepaid plans such as HMOs, or government plans such as Medicare?" All states and territories obtained health insurance information in 2002. The number of respondents analyzed for health insurance was 246,560. Medical checkups were assessed by asking: "About how long has it been since you last visited a doctor for a routine checkup?" A recent checkup was defined as having had a checkup within the past 24 months. Information about medical checkups was obtained from an optional module of questions and was collected by 10 states and territories in 2002 (Georgia, Idaho, Iowa, Maryland, Montana, New Mexico, North Dakota, South Dakota, Guam, and the U.S. Virgin Islands). The number of respondents analyzed for medical checkups was 36,987.

## Results

In 2002, 85% of the U.S. adult population reported having some form of health insurance. Individuals with current health insurance were more likely to report having had a checkup in the past 24 months compared with individuals without health insurance (88% of individuals with current health insurance vs 73% of individuals without; odds ratio, 2.70; 95% confidence interval, 2.41–3.02). Of individuals with current health insurance, 15% were excessive drinkers (i.e., reported past-month binge drinking, heavy drinking, or both); of individuals who had a recent checkup, 14% were excessive drinkers (data not shown).

Among U.S. adults, 17% were excessive drinkers, 38% were nonexcessive drinkers, and 45% were nondrinkers (data not shown). Excessive drinkers had a lower prevalence of health insurance coverage (79%) than nonexcessive drinkers (88%) and nondrinkers (84%); excessive drinkers also had a lower prevalence of medical checkups (78%) than nonexcessive drinkers (87%) and nondrinkers (88%) (Table 1). Excessive drinkers had lower rates of health insurance and checkups across all strata based on age, sex, race and ethnicity, education, income, and employment status. However, differences in health insurance coverage were less pronounced after age stratification, suggesting that young age, which is associated with both excessive drinking and a lack of health insurance, partly explains the lower rates of health insurance among excessive drinkers. Nonexcessive drinkers tended to have higher rates of insurance coverage even compared with nondrinkers, but these two groups had similar rates of checkups.

Among excessive drinkers, those who were young, male, nonwhite, less educated, unemployed, and low income had lower rates of health insurance coverage (Table 1). Among excessive drinkers, lower rates of checkups were observed among young and middle-aged adults, men, and those with less education. There were not pronounced differences in rates of checkups based on race or ethnicity, income, or employment status. Overall, 70% of excessive drinkers who lacked health insurance and 85% of excessive drinkers who lacked a recent checkup were employed (data not shown). 

Among excessive drinkers, 67% reported binge drinking but not heavy drinking; 25% reported both binge drinking and heavy drinking; and 8% reported heavy drinking but not binge drinking (data not shown). Excessive drinkers who reported both binge and heavy drinking had lower rates of health insurance (75%) and checkups (73%) compared with excessive drinkers who reported binge drinking but not heavy drinking (80% with health insurance, 79% with a checkup) or heavy drinking but not binge drinking (86% with insurance, 85% with a checkup) (Table 2). Thus, more than two thirds of the excessive drinkers who had health insurance or who had a recent checkup were binge drinkers but *not* heavy drinkers (based on average consumption).

## Discussion

Although excessive drinkers (those who reported binge drinking, heavy drinking, or both) had slightly less access to health care than nonexcessive drinkers and nondrinkers, almost 80% of excessive drinkers had current health insurance and a recent medical checkup. Furthermore, the prevalence of excessive drinking among those who had insurance (15%) or a medical checkup (14%) was only slightly lower than the national prevalence of excessive drinking (17%). These findings suggest that low rates of alcohol screening in the United States, including among individuals who drink excessively, result primarily from missed screening opportunities rather than a lack of access to preventive services. Because 92% of excessive drinkers reported binge drinking, the widespread adoption of single-question screens (which typically ask about drinking at levels that constitute binge drinking) in primary care settings could identify the vast majority of excessive drinkers in the United States while improving the acceptability of screening relative to more lengthy multiquestion instruments.

A previous study found that approximately two thirds of people with alcohol abuse or dependence had an outpatient medical visit in the preceding year (11). To our knowledge, however, this is the first national study to examine health care access among all excessive drinkers. Including all excessive drinkers is important because current screening practices attempt to identify the full spectrum of excessive drinkers, and other studies demonstrate that most people who drink excessively do not meet criteria for alcohol abuse or dependence (6). Rates of dependence are likely to be especially low among excessive drinkers who are binge drinkers but not heavy drinkers and who constituted the largest group of excessive drinkers in this study. Therefore, the majority of individuals with positive alcohol screens would be eligible for brief counseling interventions delivered in primary care settings or by telephone and would not require more expensive specialty referral as would typically be suggested for individuals with alcohol dependence. Furthermore, intervening with the large proportion of excessive drinkers who are nondependent represents an important opportunity to prevent future alcohol-related problems and costs, including those related to the development of alcohol dependence itself.

The consequences of not screening for excessive drinking extend beyond a patient's own risk of alcohol-attributable conditions; excessive drinkers are also at risk for preventable or treatable conditions (e.g., depression, anxiety, early liver disease, social problems) (12) that might be detected based on a clinician's awareness of a patient's excessive drinking. Excessive drinkers may be less compliant with treatment prescribed for chronic conditions (e.g., diabetes, hypertension, HIV) and are also at risk for complications from interactions between prescription medications and alcohol. Failure to address excessive drinking puts families and members of the general public at risk for the many secondhand effects of excessive drinking, such as interpersonal violence, motor vehicle crashes, and fetal alcohol syndrome.

Because almost one in five excessive drinkers lacked a checkup or health insurance, it is important to improve access to health care as a way of providing additional opportunities to prevent the consequences and costs of excessive drinking. One way to improve access would be to increase employer-based health insurance. Although excessive drinkers were more likely to be unemployed than nonexcessive drinkers and nondrinkers, this study demonstrated that most excessive drinkers who lacked health insurance (70%) or a recent checkup (85%) were employed. Because some of the medical and nonmedical costs due to excessive drinking are absorbed by employers (e.g., absenteeism, lost productivity), increasing employer-based health insurance coverage in general, and substance abuse coverage in particular, would reduce alcohol problems, would save costs, and could improve job performance (8,13-16).

This study had several limitations. Surveys generally, and BRFSS specifically, underestimate alcohol consumption because of a combination of inaccurate respondent recall and survey noncoverage, including noncoverage of individuals with severe alcohol problems or individuals lacking telephones (17,18). As such, it is likely that we underestimated the proportion of adults who drink excessively and who might benefit from alcohol counseling. Furthermore, if individuals not covered by the survey (including nonrespondents) had a relatively high prevalence of excessive drinking or a low prevalence of health care access, our study would have overestimated the level of health care access and health insurance coverage among excessive drinkers. However, our estimates of health insurance coverage may also underestimate access to care because those without insurance may pay out-of-pocket or may be eligible for free care. Finally, it is possible that respondents may overreport checkups (e.g., thinking that checkup meant any visit to a health professional, rather than a preventive care visit), which could lead to overestimates of the proportion of the population who accessed preventive services. However, primary care screening is not restricted to checkups, so the lack thereof does not preclude the possibility of alcohol screening.

Although BRFSS data were weighted to be representative of the United States, data about checkups were obtained in only 10 states and territories (whereas data on health insurance coverage were obtained in all states and territories). However, estimates about the prevalence of a recent checkup were likely to be nationally representative because health insurance is a strong predictor of having had a recent checkup, and the 10 states and territories that used the module had similar rates of health insurance compared with the rest of the country (86% vs 85%). In addition to providing state and national estimates, one of the strengths of BRFSS is that it can be used to assess rates of excessive drinking at the metropolitan or county level (19), which can help target clinical or public health prevention activities that focus on excessive drinking or other risk behaviors. 

Screening and counseling of adult primary care patients for alcohol misuse, including nondependent excessive drinking, is recommended by the U.S. Preventive Services Task Force (7) and is consistent with Institute of Medicine recommendations to "broaden the base" of alcohol treatment to include all types of excessive drinking, not just alcohol dependence (20). Patients with positive screens for alcohol misuse should receive a more in-depth evaluation about their alcohol consumption and its possible consequences, followed by either brief outpatient counseling or more intensive treatment for those with alcohol dependence. In primary care settings, studies have shown that brief counseling for nondependent excessive drinkers reduces total alcohol consumption and binge drinking, reduces alcohol-related health outcomes, and is cost-saving (4,8,21,22). In the absence of screening, physicians cannot reliably identify patients who drink excessively or who have alcohol problems (23).

There are multiple causes for low rates of alcohol screening by health professionals. Some barriers to screening include a lack of training about alcohol screening and brief counseling interventions, a lack of referral resources for patients identified with alcohol dependence, low compensation rates for preventive health services and counseling, clinician concerns about alienating patients, a lack of performance measures for alcohol screening and counseling, and failure to implement systems-wide approaches to encourage adherence to screening guidelines. To increase screening and counseling for alcohol problems in primary care settings, systems approaches should be developed to overcome some of these barriers. These approaches include measures such as reminder systems and the adoption of single-question screening strategies to save time for busy practices and clinicians (4). In addition, health plans should work with the National Committee for Quality Assurance to develop and implement a Health Plan Employer Data and Information Set (HEDIS) measure on screening and counseling for alcohol problems. In addition, efforts should be made to ensure uniform, reimbursable coding practices for excessive drinkers who receive brief counseling interventions. Finally, education about alcohol-related screening and counseling should be incorporated into training programs for primary care clinicians, clinic nurses, and social workers.

## Figures and Tables

**Table 1:** Health Insurance Coverage and Recent Medical Checkup^a^ Among U.S. Adults, by Alcohol Consumption Status^b^ and Selected Demographic Characteristics, Behavioral Risk Factor Surveillance System (BRFSS), 2002

**Characteristic**	**Excessive Drinkers**		**Nonexcessive Drinkers**		**Nondrinkers**	

	**Insured % (SE)**	**Checkup % (SE)**	**Insured % (SE)**	**Checkup % (SE)**	**Insured % (SE)**	**Checkup % (SE)**
**Total**	79.2 (0.43)	78.0 (1.02)	88.2 (0.21)	87.2 (0.47)	84.0 (0.23)	87.5 ( 0.42)

**Age, y**						

18-34	74.2 (0.69)	77.5 (1.64)	79.9 (0.53)	85.7 (1.02)	74.9 (0.56)	85.5 (0.96)
35-54	81.7 (0.61)	76.1 (1.43)	89.0 (0.30)	85.2 (0.76)	82.3 (0.36)	85.2 (0.74)
≥55	91.8 (0.72)	85.8 (2.39)	95.3 (0.23)	91.9 (0.59)	92.4 (0.24)	91.4 (0.54)

**Sex**						

Male	78.0 (0.55)	74.9 (1.33)	86.7 (0.34)	83.5 (0.80)	82.7 (0.40)	82.3 (0.87)
Female	82.0 (0.62)	85.7 (1.42)	89.6 (0.25)	90.6 (0.53)	84.7 (0.26)	90.8 (0.41)

**Race and ethnicity**						

White	83.4 (0.39)	76.8 (1.08)	91.8 (0.17)	86.0 (0.54)	88.3 (0.19)	86.1 (0.50)
Black	73.1 (1.61)	82.8 (3.69)	80.3 (0.90)	94.2 (1.14)	80.7 (0.64)	93.4 (0.95)
Hispanic	63.3 (1.80)	77.9 (5.24)	69.9 (1.21)	85.6 (1.88)	68.0 (0.97)	86.0 (1.43)
Other	70.4 (2.44)	84.1 (3.72)	86.7 (0.99)	84.6 (2.82)	84.6 (0.89)	84.4 (2.25)

**Education**						

≤High school	69.2 (0.76)	73.1 (1.76)	79.8 (0.48)	85.9 (0.86)	79.1 (0.35)	88.5 (0.55)
>High school	86.4 (0.47)	81.0 (1.19)	92.3 (0.21)	87.7 (0.57)	89.4 (0.26)	86.4 (0.65)

**Employment**						

Employed	80.6 (0.49)	75.9 (1.12)	88.8 (0.26)	85.7 (0.59)	83.6 (0.31)	85.7 (0.61)
Unemployed	55.4 (1.72)	76.7 (4.41)	68.5 (1.12)	89.2 (1.99)	73.4 (0.78)	89.4 (1.35)
Student, homemaker, retired	86.1 (0.92)	89.1 (2.04)	92.3 (0.33)	91.0 (0.76)	88.3 (0.34)	89.8 (0.62)

**Annual income, $**						

<25,000	60.2 (1.09)	78.7 (2.35)	69.5 (0.75)	85.0 (1.25)	74.5 (0.48)	86.6 (0.70)
25,000–50,000	79.0 (0.76)	75.4 (1.79)	87.6 (0.38)	86.9 (0.82)	87.0 (0.36)	87.6 (0.77)
>50,000	92.4 (0.52)	79.0 (1.58)	96.8 (0.16)	87.6 (0.73)	94.9 (0.28)	87.6 (0.91)

a A recent medical checkup was defined as having had a routine checkup during the past 24 months. Data on checkups were obtained from 10 states and territories (n = 36,987). Data on health insurance were obtained from 53 states and territories (n = 246,560).

b Excessive drinkers were defined as men who reported drinking five or more drinks on at least one occasion or who consumed more than 60 drinks during the past month and women who drank five or more drinks on at least one occasion or who consumed more than 30 drinks during the past month. Nonexcessive drinkers were those who reported consuming some alcohol during the past 30 days but who did not drink excessively. Nondrinkers reported no alcohol consumption during the past 30 days.

**Table 2:** Health Insurance Coverage and Recent Medical Checkup Among U.S. Adults Who Drink, by Pattern of Alcohol Consumption, Behavioral Risk Factor Surveillance System (BRFSS), 2002

**Drinking Pattern^a^**	**Insured, % (SE)**	**Checkup^b^, % (SE)**

**Excessive**		

Binge, heavy	74.8 (0.89)	72.6 (2.05)
Binge, nonheavy	80.1 (0.53)	79.1 (1.24)
Nonbinge, heavy	86.4 (1.16)	84.5 (3.27)

**Nonexcessive**		

Nonbinge, nonheavy	88.2 (0.21)	87.2 (0.47)

a Binge drinkers were respondents who reported drinking five or more drinks on at least one occasion during the past month. Heavy drinkers were men who drank more than 60 drinks during the past month or women who drank more than 30 drinks during the past month.

b A recent medical checkup was defined as having had a routine checkup during the past 24 months. Data on checkups were obtained from 10 states and territories (n = 36,987).
